# Socioeconomic and demographic inequalities in stage at diagnosis and survival among colorectal cancer patients: evidence from a Swiss population‐based study

**DOI:** 10.1002/cam4.1385

**Published:** 2018-02-26

**Authors:** Anita Feller, Kurt Schmidlin, Andrea Bordoni, Christine Bouchardy, Jean‐Luc Bulliard, Bertrand Camey, Isabelle Konzelmann, Manuela Maspoli, Miriam Wanner, Marcel Zwahlen, Kerri M. Clough‐Gorr, the SNC and the NICER Working Group, Matthias Egger, Adrian Spoerri, Milo Puhan, Matthias Bopp, Nino Künzli, Murielle Bochud, Michel Oris

**Affiliations:** ^1^ Institute of Social and Preventive Medicine (ISPM) University of Bern Finkenhubelweg 11 CH‐3012 Bern Switzerland; ^2^ National Institute for Cancer Epidemiology and Registration (NICER) Hirschengraben 82 8001 Zürich Switzerland; ^3^ Ticino Cancer Registry Instituto cantonale di patologia Via in Selva 24 6601 Locarno 1 Switzerland; ^4^ Geneva Cancer Registry Institute of Global Health University of Geneva Bd de la Cluse 55 1205 Geneva Switzerland; ^5^ Vaud Cancer Registry University Institute of Social and Preventive Medicine (IUMSP) Route de la Corniche 10, Bâtiment Biopôle 2 1010 Lausanne Switzerland; ^6^ Fribourg Cancer Registry St. Nicolas de Flüe 2 1705 Fribourg Switzerland; ^7^ Health Observatory Valais Valais Cancer Registry Avenue Grand‐Champsec 64 1950 Sion Switzerland; ^8^ Neuchâtel and Jura Cancer Registry Rue du Plan 30 2000 Neuchâtel Switzerland; ^9^ Cancer Registry Zurich and Zug Biostatistics and Prevention Institute University Zurich Vogelsangstrasse 10 8091 Zurich Switzerland; ^10^ National Cancer Registry Ireland Airport Business Park 6800 Cork Ireland

**Keywords:** Colorectal cancer, health inequalities, socioeconomic position, stage at diagnosis, survival

## Abstract

Socioeconomic inequalities in cancer stage at diagnosis and survival are important public health issues. This study investigates the association between socioeconomic position (SEP) and colorectal cancer (CRC) stage at diagnosis and survival in Switzerland, a European country with highest level of medical facilities and life expectancy. We used population‐based CRC data from seven Swiss cantonal cancer registries 2001–2008 (*N* = 10,088) linked to the Swiss National Cohort (SNC). Follow‐up information was available until the end of 2013. SEP was estimated based on education. The association between cancer stage and SEP was assessed using logistic regression models including cancer localization (colon/rectum), sex, age, civil status, urbanity of residence, language region, and nationality (Swiss/non‐Swiss). Survival was analyzed using competing risk regressions reporting subhazard ratios (SHRs) for the risk of dying due to CRC. We observed a social gradient for later stage CRC with adjusted odds ratios (ORs) of 1.11 (95% CI: 0.97–1.19) and 1.28 (95% CI: 1.08–1.50) for middle and low SEP compared to high SEP. Further, single compared to married people had elevated odds of being diagnosed at later stages. Survival was lower in patients with CRC with low SEP in the unadjusted model (SHR: 1.18, 95% CI: 1.07–1.30). After adjustment for stage at diagnosis and further sociodemographic characteristics, significant survival inequalities by SEP disappeared but remained for non‐Swiss compared to Swiss citizens and for patients living in nonurban areas compared to their urban counterparts. Swiss public health strategies should facilitate equal access to CRC screening and optimal CRC care for all social groups and in all regions of Switzerland.

## Background

In Switzerland, colorectal cancer (CRC) is the third most common cancer in men and the second most common in women. Approximately 2300 men and 1800 women are newly diagnosed with CRC each year 1. Although mortality decreased over the last 30 years, CRC is the third most common cause of cancer death among both sexes in Switzerland with approximately 900 deaths among men and 700 among women 1. Tumor stage at diagnosis is an important prognostics factor, and survival is generally good for patients with early‐stage CRC 2. Although Switzerland has one of the highest survival rate of CRC in Europe 3, the 5‐year relative survival in Switzerland ranges from 12.9% (UICC stage IV) to 93.3% (UICC stage I) depending on stage at diagnosis 4. Therefore, early CRC detection is essential to increase treatment options and improve outcome. However, most early‐stage CRCs produce no clinical symptoms and thus are generally diagnosed in individuals undergoing screening 5, 6.

In Switzerland, health care and preventive services are organized at the cantonal level. Up to now, population‐based organized CRC screening has only been implemented in Vaud, one of 26 cantons in Switzerland, starting in 2015, and offering fecal immunological test or colonoscopy to people aged 50–69 years 7. The canton of Uri maintains an organized program since 2000, but the target population is not individually invited to attend screening. In the remaining cantons, only opportunistic CRC screening is available, although the benefit of CRC screening is well accepted 8, 9.

A negative impact of low socioeconomic position (SEP) on cancer stage at diagnosis has been documented for several cancer sites including CRC 10, 11, 12. Later stage at CRC diagnosis in lower SEP patients might be mediated by disparities in healthcare access 13, cancer awareness 14, and/or beliefs and attitudes toward cancer and preventive services such as screening 15. While CRC screening prevalence is generally low in Switzerland (22% in 2012 in residents aged 50–75 years) 16, persisting socioeconomic inequalities have been reported such that individuals with higher SEP use CRC screening more frequently 16, 17. Further, screening prevalence by geographic residence in Switzerland changed over time with a shift from higher (year 2007) to lower (year 2012) screening prevalence in the rural compared to the urban population 16, 17.

Worse survival in low SEP patients after CRC diagnosis has been observed in several countries 18. In most studies 12, 19, 20, 21, survival inequalities remained after adjustment for stage at diagnosis and other prognostic factors suggesting that additional factors such as quality of care, treatment preferences, and/or adherence might have contributed to observed survival discrepancies 12.

Improving health in the whole population requires a focus on social equity 22. However, despite high health expenditures, universal health insurance coverage, and one of the highest life expectancies in the world 23, there is recent evidence of socioeconomic and sociodemographic inequalities in cancer detection and survival in Switzerland 24. Up to now, there is no population‐based investigation of this important issue for CRC in Switzerland. Therefore, this study aimed to investigate socioeconomic and demographic inequalities in CRC stage at diagnosis and survival in a Swiss population‐based sample of patients with CRC diagnosed between 2001 and 2008.

## Patients and Methods

### Data sources

We used data from the SNC‐NICER Cancer Epidemiology Study. The SNC‐NICER Cancer Epidemiology Study is a historical cohort created to investigate sociodemographic factors associated with cancer burden in Switzerland. Within the framework of the study, the Swiss National Cohort (SNC) has been probabilistically linked to incidence data of seven Swiss cancer registries (CRs) organized in the National Institute for Cancer Epidemiology and Registration (NICER) cancer registry network.

An in‐depth description of the SNC can be found elsewhere 25. Briefly, census data of 1990 and 2000 were probabilistically linked to emigration or cause‐specific mortality records of the years 1991–2013. The Swiss census is mandatory and virtually complete (98.6% in the 2000 census) 25. The coding of death certificates and the selection of the underlying cause of death are carried out by trained staff of the Federal Statistical Office (FSO) for the whole country. Since 1995, the 10th revision of the International Classification of Diseases and related health problems (ICD‐10) has been used for cause of death coding following international standards. This study used SNC sociodemographic information on education level, marital status, urbanity of residence, language region of residence, nationality, and SNC follow‐up information up to the end of 2013. The categorization in urban, periurban, and rural is based on the spatial classification of communities as defined by the Swiss Federal Statistical Office (SFSO). The SFSO classifies each community as (1) isolated city, (2) main city of an agglomeration, (3) other agglomeration community, and (4) rural community. A detailed description can be found elsewhere 26. For this study, isolated cities and main cities of agglomerations have been categorized as “urban,” other agglomeration communities as “periurban,” and rural communities as “rural”.

Cancer registration in Switzerland is primarily organized at the cantonal level. The cantonal CRs record all incident cancer cases diagnosed in their resident population. Population‐based cancer registration started in Switzerland in 1970 and has since been gradually implemented in 23 of 26 cantons, with full national coverage planned for the year 2019. However, some of these CR just started recently and were therefore not eligible for this study. A detailed description of the history and organization of cancer registration in Switzerland can be found elsewhere 27.

For the SNC‐NICER Cancer Epidemiology Study, all CRs implemented before 2008 were invited to participate. Seven of 11 CRs eligible for the study provided incidence data for the study, starting from the date of Census 1990 (or earliest year available if later) through the end of 2008: Fribourg (2006–2008), Geneva (1990–2008), Neuchâtel (1990–2008), Ticino (1996–2008), Valais (1990–2008), Vaud (1990–2008), and Zurich (1990–2008). In 2008, these cantons represented 46.1% of the Swiss population.

Cancer registries cases linked to the SNC included all primary cancers diagnosed at or after Census 1990 (or earliest year available for CRs implemented after 1990) through the end of 2008. Cancer registration information comprised sex, date of birth, date of cancer diagnosis, basis of diagnosis, topography, morphology, and behavior of the tumor (according to the International Classification of Diseases for Oncology, third edition (ICD‐O‐3)), and Tumour, Node and Metastasis staging information (TNM, 5th or 6th edition).

### Study population

The final study population included 10,088 invasive colorectal cancer cases diagnosed between Census 2000 (5 December 2000) and 31 December 2008. Preceding time periods were excluded from analyses due to high proportions of missing stage information (overall >30%, >90% for single years in two cantons). People diagnosed at 85 years of age or older were excluded (*N* = 1478) because of comparably low completeness of stage information (72.7%) and data quality (percentage of histologically verified cases: 83.9%; death certificate only percentage DCO%: 5.4%) in this age‐group. Highest completed education level was used as a proxy for SEP; hence, people under 30 years of age at diagnosis (*N* = 38) and people with missing education information (*N* = 86) were excluded from the study population. The study population showed DCO% of 0.8% indicating high completeness of case ascertainment. Nearly 98% of the cases were histologically verified, and 90.1% had sufficient TNM information to classify tumor stage.

To investigate sample representativeness, we compared sample characteristics between residents of participating cantons and all Switzerland using sociodemographic information from the Census 2000 (age, sex, civil status, education, urbanity, and language region of residence) (Table S1). For the age range included in this study (30–84 years), the participating cantons compared to all Switzerland showed a slightly different SEP distribution with 24.6% and 22.2% classified as high SEP and 48.5% and 51.3% classified as middle SEP, respectively. Clear differences in the distribution were observed for urbanity of residence, language region, and nationality (Table S1).

### Statistical methods

Socioeconomic position was classified as low SEP (compulsory education or less), middle SEP (secondary education), and high SEP (tertiary education).

For stage calculation, we gave priority to pathological *T* and *N* over clinical *T* and *N*. If clinical and pathological M was present, any indication of metastasis was prioritized. Missing M and Mx were categorized as M0.

We calculated UICC stage I‐IV depending on the TNM edition used for coding (5th and 6th edition). Coding changes between the 5th and the 6th edition impacted subclassifications (such as IIIa, IIIb.), but not the broader categories used within this study.

To assess the association between cancer stage at diagnosis and SEP, we used logistic regression models with the dependent variable being the dichotomized stage of the CRC (UICC stage I vs. more advanced stages) for our main analysis, complemented by recalculations using alternative cutoffs (stage I‐II vs. stage III‐IV and stage I‐III vs. stage IV) The cutoff was chosen based on evidence that individuals with stage I CRC are more likely asymptomatic than individuals with stage II‐IV CRCs and, therefore, are most likely detected through screening 28, 29. In addition, the prognostic value of UICC stage is limited for intermediate stages (stage II and stage III) 2. The term “later stages” refers to the cutoff stage I versus stage II‐IV throughout the manuscript if not stated otherwise.

We included all variables of interest available in either the CR or SNC dataset. We calculated five models using the following variables as predictors for CRC stage at diagnosis: (model 1) SEP; (model 2) model 1 plus age at diagnosis (30–49, 50–64, 65–74, and 75–84 years), civil status (single, married, widowed, and divorced), and nationality (Swiss and non‐Swiss); (model 3) model 2 plus urbanity (urban, periurban, and rural) and language region (German‐, French‐, and Italian‐speaking region); (model 4) model 3 plus tumor localization (colon and rectum); and (model 5) model 4 plus canton of residence. Results were reported as odds ratios (OR) with 95% confidence intervals (95% CI).

To analyze survival, we performed competing risk regressions based on Fine and Gray's proportional hazard model as main analysis 30. We used a competing risk approach because we were mainly interested in estimating true differences in CRC mortality risks (“real world setting”) rather than estimating hazard ratios for a hypothetical world where competing risks do not exist. However, this approach considers both hazards—the hazard due to CRC death and the hazard due to other causes of death—conjointly. Therefore, we additionally calculated cause‐specific hazard ratios (CHRs) for CRC death and other causes of death using Cox proportional hazard models.

For our main analysis, all underlying causes of death other than CRC were classified as competing risks. We used six models including the following covariates: (model 1) SEP; (model 2) model 1 plus age at diagnosis, civil status, and nationality; (model 3) model 2 plus urbanity and language region of residence; (model 4) model 3 plus tumor localization; (model 5) model 4 plus UICC stage at diagnosis; and (model 6) model 5 plus canton of residence. Results were reported as subhazard ratios (SHRs) for the risk of dying due to CRC with associated 95% confidence intervals (95% CI).

The final models have been additionally adjusted for canton of residence to account for unmeasured canton characteristics associated with SEP and stage and/or survival. We checked the influence of year of diagnosis in preliminary analysis, but observed only marginal changes after considering it. Therefore, calendar year has not been considered in the final models.

### Sensitivity analyses

To assess whether missing stage information biased our results, we recalculated all final models after multiple imputation of stage at diagnosis with 25 imputations 31. UICC stage was imputed using the following variables as predictors: age at diagnosis and follow‐up time as continuous predictors, SEP, sex, civil status, nationality, urbanity, language region, tumor localization, and follow‐up status (alive, death due to CRC, and other causes of death) as categorical predictors 32, and an interaction term between follow‐up time and follow‐up status 33.

## Results

The contribution of incident CRC cases (*N*_total _= 10,088, *N*_staged _= 9147) and person‐years (PY) of follow‐up (PYs_total _= 56,657; PY_staged _= 52,514) to the final study population by CR is presented in Table S2. Overall, around 30% of the patients with CRC belonged to the low SEP group and 50% to the middle SEP group. A summary of patient characteristics by SEP is presented in Table 1.

**Table 1:** Characteristics of patients with colorectal cancer by socioeconomic position (SEP)

Analysis of SEP and stage at diagnosis	Low SEP		Middle SEP		High SEP		Total	
	*N*	Column %	*N*	Column %	*N*	Column %	*N*	Column %
Sex								
Male	1215	40.9	2892	57.6	1593	76.0	5700	56.5
Female	1757	59.1	2127	42.4	504	24.0	4388	43.4
Age at diagnosis								
<50 years	135	4.5	361	7.2	172	8.2	668	6.6
50–64 years	672	22.6	1570	31.3	765	36.5	3007	29.8
65–74 years	890	30.0	1617	32.2	673	32.1	3180	31.5
75–84 years	1275	42.9	1471	29.3	487	23.2	3233	32.0
Civil status								
Single	259	8.7	453	9.0	213	10.2	925	9.2
Married	1851	62.3	3404	67.8	1523	72.6	6778	67.2
Widowed	617	20.8	619	12.3	132	6.3	1368	13.6
Divorced	245	8.2	543	10.8	229	10.9	1017	10.1
Urbanity of residence								
Urban	1109	37.3	1728	34.4	693	33.1	3530	35.0
Periurban	1271	42.8	2604	51.9	1182	56.4	5057	50.1
Rural	592	19.9	687	13.7	222	10.6	1501	14.9
Language region of residence^a^								
German‐speaking region	940	31.6	2288	45.6	852	40.6	4080	40.4
French‐speaking region	1571	52.9	2120	42.2	1034	49.3	4725	46.8
Italian‐speaking region	461	15.5	611	12.2	211	10.1	1283	12.7
Nationality								
Swiss	2183	73.5	4383	87.3	1757	83.8	8323	82.5
Non‐Swiss	789	26.6	636	12.7	340	16.2	1765	17.5
Localization of the cancer								
Colon	1971	66.3	3260	65.0	1344	64.1	6575	65.2
Rectum	1001	33.7	1759	35.1	753	35.9	3513	34.8
Stage at diagnosis (UICC stage)								
Stage I	434	14.6	847	16.9	385	18.4	1666	16.5
Stage II	827	27.8	1347	26.8	521	24.9	2695	26.7
Stage III	868	29.2	1383	27.6	582	27.8	2833	28.1
Stage IV	564	19.0	991	19.7	398	19.0	1953	19.4
Unknown stage	279	9.4	451	9.0	211	10.1	941	9.3
Vital status at end of follow‐up								
Alive	1794	60.4	3288	65.5	1448	69.1	6530	64.7
Dead	1152	38.8	1718	34.2	645	30.8	3515	34.8
Lost to follow‐up	26	0.9	13	0.3	4	0.2	43	0.4
Total *N* row %	2972	29.5	5019	49.8	2097	20.8	10,088	100.0

a German‐speaking region: eastern parts of the canton of Fribourg, eastern parts of the canton of Valais (upper Valais), and canton of Zurich; French‐speaking region: western parts of the canton of Fribourg, canton of Geneva, canton of Neuchâtel, western parts of the canton of Valais (Central and Lower Valais), and canton of Vaud; Italian‐speaking region: canton of Ticino.

### Colorectal cancer stage at diagnosis

Almost two‐thirds (65%) of all CRC cases were primarily located in the colon and 35% in the rectum (Table 1). The majority of all CRC cases were diagnosed at UICC stage III (28.1%) or stage II (26.7%). Only 16.5% of all cases were detected at stage I. In 9.3% of all cases, stage information was missing. The distribution by stage was fairly stable over time.

**Figure 1:**
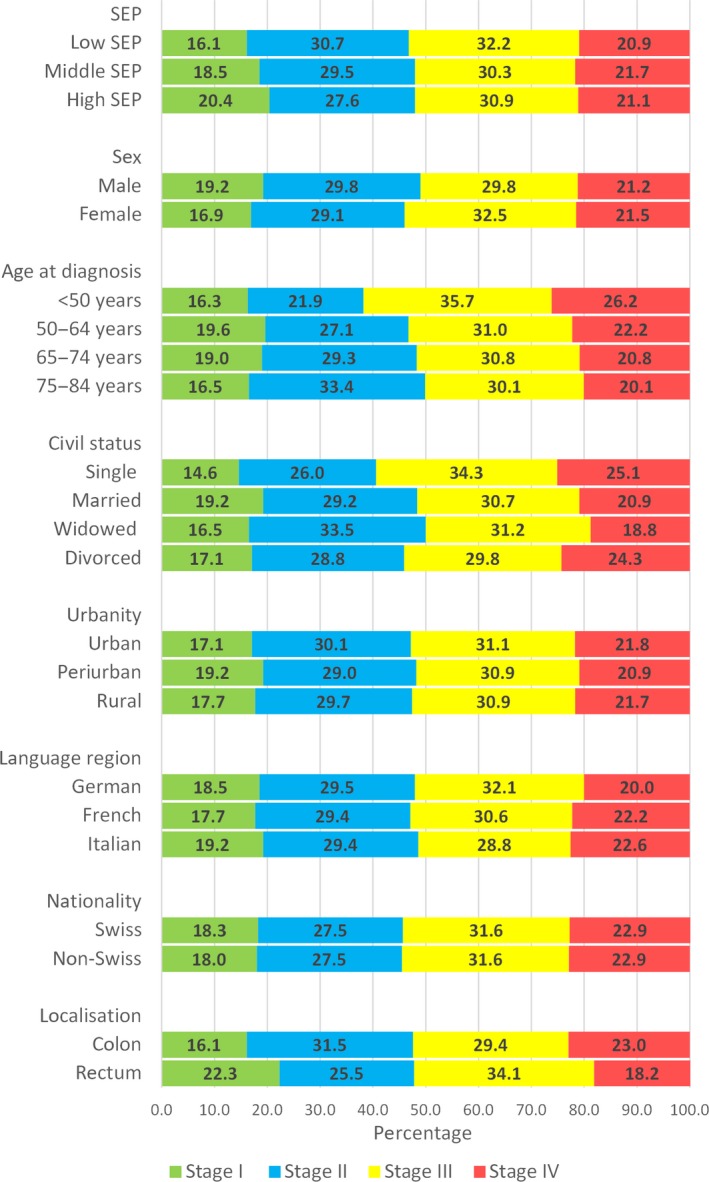
Distribution of colorectal cancer stage at diagnosis by sociodemographic characteristics and tumor localization (*N* = 9147). The analysis has been restricted to cases with known stage at diagnosis (9147 of 10,088 cases).

The stage distribution at diagnosis for each analytic variable (SEP, further sociodemographic factors and tumor localization) is presented in Figure 1.

In the unadjusted model (model 1), we observed a social gradient for later stage at diagnosis with ORs of 1.13 (95% CI: 0.99–1.29) and 1.35 (95% CI: 1.16–1.57) for middle and low SEP patients compared to high SEP patients, respectively (Table 2). After adjustment for further demographic factors, tumor localization, and canton of residence (model 5), socioeconomic inequalities slightly decreased to 1.11 (95% CI: 0.97–1.27) for middle SEP and to 1.28 (95% CI: 1.08–1.50) for low SEP. However, we observed no social gradients for the alternative cutoffs contrasting stage I‐II versus stage III‐IV (Table S3) and stage I‐III versus stage IV (Table S4).

**Table 2:** Odds ratios (OR) of later colorectal cancer stage at diagnosis (stage I vs. stage II–IV)

	Model 1	Model 2	Model 3	Model 4	Model 5^a^
	OR [95% CI]	OR [95% CI]	OR [95% CI]	OR [95% CI]	OR [95% CI]
SEP					
High SEP (ref.)					
Middle SEP	1.13 [0.99–1.29]	1.11 [0.97–1.27]	1.11 [0.97–1.27]	1.12 [0.97–1.28]	1.11 [0.97–1.27]
Low SEP	1.35 [1.16–1.57]	1.28 [1.09–1.50]	1.27 [1.08–1.50]	1.29 [1.09–1.51]	1.28 [1.08–1.50]
Sex					
Male (ref.)					
Female		1.08 [0.96–1.21]	1.08 [0.96–1.21]	1.05 [0.93–1.18]	1.05 [0.93–1.18]
Age at diagnosis					
50–64 years (ref.)					
<50 years		1.22 [0.96–1.54]	1.22 [0.96–1.55]	1.22 [0.96–1.54]	1.22 [0.96–1.54]
65–74 years		1.03 [0.90–1.18]	1.03 [0.91–1.18]	1.02 [0.89–1.16]	1.02 [0.89–1.16]
75–84 years		1.20 [1.04–1.38]	1.19 [1.03–1.38]	1.14 [0.98–1.31]	1.14 [0.99–1.32]
Civil status					
Married (ref.)					
Single		1.36 [1.11–1.68]	1.35 [1.10–1.66]	1.35 [1.10–1.67]	1.36 [1.10–1.67]
Widowed		1.05 [0.88–1.25]	1.04 [0.87–1.25]	1.05 [0.88–1.25]	1.05 [0.88–1.25]
Divorced		1.16 [0.97–1.39]	1.15 [0.96–1.38]	1.15 [0.96–1.38]	1.15 [0.96–1.38]
Nationality					
Swiss (ref.)					
Non‐Swiss		1.02 [0.88–1.18]	1.02 [0.88–1.18]	1.02 [0.88–1.18]	1.03 [0.88–1.19]
Urbanity					
Urban (ref.)					
Periurban			0.92 [0.82–1.04]	0.92 [0.82–1.04]	0.93 [0.82–1.04]
Rural			0.98 [0.82–1.16]	0.97 [0.81–1.15]	0.96 [0.80–1.15]
Language region^b^					
German (ref.)					
French			1.04 [0.93–1.17]	1.04 [0.92–1.17]	1.19 [0.84–1.69]
Italian			0.95 [0.81–1.13]	0.93 [0.79–1.10]	0.93 [0.78–1.10]
Localization					
Colon (ref.)					
Rectum				0.68 [0.61–0.76]	0.68 [0.61–0.76]

a Additionally adjusted for canton of residence to account for unmeasured canton characteristics associated with SEP and stage.

b German‐speaking region: eastern parts of the canton of Fribourg, eastern parts of the canton of Valais (upper Valais), and canton of Zurich; French‐speaking region: western parts of the canton of Fribourg, canton of Geneva, canton of Neuchâtel, western parts of the canton of Valais (Central and Lower Valais), and canton of Vaud; Italian‐speaking region: canton of Ticino.

Concerning civil status, we observed an increased risk of later stage at CRC diagnosis for single compared to married patients (OR 1.36, 95% CI: 1.10–1.67), but not for widowed or divorced ones. Further, rectal cancer was less likely to be diagnosed at later stage compared to colon cancer (OR 0.68, 95% CI: 0.61–0.76).

### Colorectal cancer survival

Of the 10,088 patients with CRC, 3515 died before the end of follow‐up (34.8%) and 43 patients (0.4%) were lost to follow‐up (Table 1).

Patients with CRC with low SEP were more likely to die of their disease compared to patients with CRC with high SEP in the unadjusted model (SHR 1.18, 95% CI: 1.07–1.30) (Table 3, model 1). After adjustment for further demographic characteristics, relative mortality risk of low SEP patients shrank to SHR 1.14 (95% CI: 1.02–1.27) (model 3) and declined further to SHR 1.08 (95% CI: 0.96–1.21) (model 6) after additional adjustment for stage at diagnosis, tumor localization, and canton of residence. Later stage at diagnosis was strongly associated with an increased risk of CRC death (stage II: SHR 3.20, 95% CI: 2.54–4.02; stage III: SHR 8.13, 95% CI: 6.54–10.10; stage IV: SHR 30.00, 95% CI: 24.15–27.21 compared with patients diagnosed with stage I CRCs). Further, patients with rectal cancer compared with patients with colon cancer (SHR 1.10, 95% CI: 1.01–1.19), patients with foreign nationality compared with Swiss citizens (SHR 1.20, 95% CI: 1.08–1.34), and patients living in a periurban (SHR 1.15, 95% CI: 1.05–1.25) or rural area (SHR 1.15, 95% CI: 1.02–1.30) compared with patients living in urban areas showed an elevated risk of CRC death in the fully adjusted model. Patients diagnosed below the age of 50 years showed the most favorable survival (SHR 0.69, 95% CI: 0.58–0.82), followed by patients aged 50–64 years (reference group) and patients aged 65–74 years (SHR 1.17, 95% CI: 1.07–1.29). Worst survival was observed for patients aged 75–84 years at diagnosis, the oldest age‐group included in this study, with a SHR of 1.68 (95% CI: 1.51–1.85) compared with patients diagnosed at age 50–64 years).

**Table 3:** Subhazard ratios and 95% confidence intervals (95% CI), risk of colorectal cancer death in patients with colorectal cancer

	Model 1	Model 2	Model 3	Model 4	Model 5	Model 6^a^
	SHR [95% CI]	SHR [95% CI]	SHR [95% CI]	SHR [95% CI]	SHR [95% CI]	SHR [95% CI]
SEP						
High SEP (ref.)						
Middle SEP	1.07 [0.98–1.17]	1.05 [0.96–1.16]	1.05 [0.95–1.15]	1.05 [0.95–1.15]	0.99 [0.90–1.10]	0.99 [0.89–1.09]
Low SEP	1.18 [1.07–1.30]	1.14 [1.02–1.27]	1.14 [1.02–1.27]	1.13 [1.02–1.26]	1.09 [0.97–1.22]	1.08 [0.96–1.21]
Sex						
Male (ref.)						
Female		0.96 [0.88–1.03]	0.96 [0.89–1.04]	0.96 [0.89–1.04]	0.95 [0.88–1.03]	0.95 [0.88–1.04]
Age at diagnosis						
50–64 years (ref.)						
<50 years		0.80 [0.68–0.95]	0.80 [0.68–0.94]	0.80 [0.68–0.95]	0.69 [0.59–0.83]	0.69 [0.58–0.82]
65–74 years		1.11 [1.01–1.21]	1.11 [1.02–1.22]	1.11 [1.02–1.22]	1.18 [1.07–1.29]	1.17 [1.07–1.29]
75–84 years		1.48 [1.35–1.62]	1.49 [1.36–1.64]	1.49 [1.36–1.64]	1.67 [1.51–1.85]	1.68 [1.51–1.85]
Civil status						
Married (ref.)						
Single		1.14 [1.00–1.29]	1.15 [1.01–1.31]	1.14 [1.01–1.30]	0.97 [0.85–1.12]	0.99 [0.86–1.14]
Widowed		0.89 [0.79–1.00]	0.89 [0.79–1.00]	0.89 [0.79–1.00]	0.95 [0.83–1.08]	0.95 [0.84–1.08]
Divorced		1.10 [0.98–1.24]	1.11 [0.99–1.25]	1.10 [0.98–1.24]	1.00 [0.88–1.13]	1.02 [0.90–1.15]
Nationality						
Swiss (ref.)						
Non‐Swiss		1.15 [1.04–1.27]	1.13 [1.02–1.25]	1.13 [1.02–1.25]	1.22 [1.10–1.36]	1.20 [1.08–1.34]
Urbanity						
Urban (ref.)						
Periurban			1.08 [1.00–1.17]	1.07 [1.00–1.17]	1.13 [1.04–1.23]	1.15 [1.05–1.25]
Rural			1.16 [1.04–1.29]	1.15 [1.04–1.29]	1.15 [1.03–1.30]	1.15 [1.02–1.30]
Language region^b^						
German (ref.)						
French			0.95 [0.88–1.03]	0.95 [0.88–1.03]	0.93 [0.85–1.01]	0.97 [0.77–1.23]
Italian			0.93 [0.83–1.04]	0.93 [0.83–1.04]	0.90 [0.80–1.02]	0.91 [0.81–1.03]
Localization						
Colon (ref.)						
Rectum				1.01 [0.941.09]	1.10 [1.01–1.19]	1.10 [1.01–1.19]
Stage at diagnosis						
Stage I (ref.)						
Stage II					3.20 [2.55–4.02]	3.20 [2.54–4.02]
Stage III					8.11 [6.53–10.08]	8.13 [6.54–10.10
Stage IV					29.83 [24.03–37.03]	30.00 [24.15–37.21]

Survival was analyzed using competing risk regressions based on Fine and Gray's proportional hazard model. All underlying causes of death other than colorectal cancer (CRC) were classified as competing risks. Results are reported as subhazard ratios for risk of dying due to CRC (SHRs) with 95% confidence intervals (95% CI).

a Additionally adjusted for canton of residence to account for unmeasured canton characteristics associated with SEP and stage and/or survival.

b German‐speaking region: eastern parts of the canton of Fribourg, eastern parts of the canton of Valais (upper Valais), and canton of Zurich; French‐speaking region: western parts of the canton of Fribourg, canton of Geneva, canton of Neuchâtel, western parts of the canton of Valais (Central and Lower Valais), and canton of Vaud; Italian‐speaking region: canton of Ticino.

Cause‐specific hazard ratios of CRC death and other causes of death in patients diagnosed with CRC are presented in Table S5. The factors found to have significant association with CRC death were identical in both approaches with similar effect sizes in both final models.

### Sensitivity analyses

Stage at presentation and survival analyses after multiple imputation of stage information are presented in Tables S6 and S7, respectively. The multiple imputation approach showed similar results compared to our main analyses using listwise deletion (complete case analyses) to handle missing stage information. Overall, there is no evidence for biased estimates or insufficient precision due to our approach using listwise deletion.

## Discussion

### Summary of main findings

Our study provides evidence for socioeconomic inequalities in CRC stage at diagnosis and subsequent survival in Switzerland. Differences in survival were mainly driven by inequalities in stage at diagnosis rather than stage‐specific survival inequalities. Compared with Swiss nationals and urban residents, foreign residents and those living in urban or periurban areas had poorer prognosis, even after adjustment for stage at diagnosis, SEP, and further sociodemographic characteristics.

### Discussion in the context of the literature

Studies conducted in other developed countries have reported socioeconomic inequalities in CRC stage at diagnosis 10, 11, 12 and survival 11, 18, 19, 20. However, social disparities were not observed for all regions or countries and for all time periods investigated 34, 35. A study based on Surveillance, Epidemiology and End Results (SEER) data 1973–2001, for example, reported socioeconomic stage disparities for breast and prostate cancer, but not for CRC 35. In another population‐based study conducted in Portugal, socioeconomic inequalities in CRC survival disappeared after accounting for differences in background mortality 34. In our study, socioeconomic inequalities in stage at diagnosis appeared to be the main determinant of inequalities in CRC survival.

Inequalities in stage at diagnosis across social groups are usually explained by disparities in healthcare access 13, cancer awareness 14, and/or beliefs and attitudes toward cancer and preventive services such as screening 15. However, the importance of each of these factors can be expected to vary by country/region and healthcare system. Disparities in healthcare access, for example, might be of lesser importance in countries with national health insurance compared to countries with strictly voluntary or private health insurance. Therefore, underlying mechanisms for these social inequalities have to be investigated on a national or regional basis.

In our study, socioeconomic inequalities in stage at diagnosis decreased after adjustment for further demographic characteristics, but did not fully disappear. In addition, a social gradient was only observed for the main cutoff contrasting stage I versus stage II‐stage IV, but not for the alternative cutoffs. In the descriptive analysis, stage differences between SEP groups appeared rather small and were most pronounced for stage I and stage II CRCs, whereas proportions of stage III and metastatic CRC were similar across SEP groups. Early‐stage CRCs are generally asymptomatic and mainly diagnosed through screening 5, 6. Therefore, observed disparities in stage at diagnosis might be best explained by differences in screening activities. Two recent Swiss studies showed that individuals of lower SEP were less likely to undergo CRC screening 16, 17. In 2012, for example, CRC screening utilization in Switzerland in individuals aged 50–75 years was 28.6% and 16.0% for people in the highest and lowest income quintile, respectively 16.

For the time period under investigation, no organized population‐based CRC screening program existed in Switzerland. Whereas organized screening programs are now widespread in Europe 36, 37, only one canton in Switzerland has to date implemented a population‐based CRC screening program. Despite universal health insurance in Switzerland, CRC screening was not free in the time period under investigation. Only since July 2013, two methods of CRC screening (fecal occult blood test and colonoscopy) have been added to basic health coverage. But importantly, CRC screening has not been exempted from the franchise and the deductible. Reimbursement regardless of franchise is currently only guaranteed in the cantons of Vaud and Uri covering around 10% of the Swiss population. Therefore, the majority of individuals with only basic health insurance have still to cover the full costs in case the annual franchise (300–2500 CHF) is not met (or up to 10% of the costs as a deductible if the annual upper limit of 700 CHF has not been reached).

We observed a significant increased risk of later stage CRC at diagnosis for single compared to married patients. An impact of marital status on cancer stage at diagnosis, treatment, and/or survival has been reported in other studies 24, 38, 39, 40. A SEER study, for example, found that unmarried compared to married patients were at higher risk of being diagnosed with metastatic cancer, undertreatment, and cancer death, suggesting that decreased social support may negatively influence cancer detection, treatment uptake, and survival 38. However, in our study, an increased risk of being diagnosed at later stages was only seen in single patients. This was reflected by the lower overall survival of single compared to married patients. After stage adjustment, observed survival disparities did not persist, suggesting that treatment uptake and quality of care did not depend on civil status.

Looking at the age at diagnosis, patients with CRC below the age of 50 years had the most favorable overall and stage‐adjusted survival, although they had the highest proportion of stage III and stage IV cancers. Better or equal prognosis of younger than older patients, even when presenting at later stages and with more aggressive tumors, has been previously reported 41, 42, 43.

The survival advantage of younger patients might be associated with less comorbidities, more aggressive treatment, better treatment adherence, and/or less treatment‐associated complications. Notably, there is also evidence from the United States that young patients with colon cancer are overtreated and at an increased risk to receive unnecessary chemotherapy 44. For older patients, an opposite trend has been reported suggesting that older patients with CRC (>75 years) are often undertreated 45. The oldest patients included in our study (75–84 years) clearly experienced the poorest survival. Further investigations are needed to disentangle the impact of patient‐, tumor‐, and treatment‐related factors on age‐specific outcomes in Swiss patients with CRC.

Patients living in periurban and rural areas showed lower stage‐adjusted survival compared with their urban counterparts. Some studies from other countries reported similar results for patients with CRC 46, 47, but other studies found the opposite result 48. Although Switzerland has a high hospital density 23, inhabitants of remote regions, peripheral valleys, or mountainous areas may have long travel distances for care. Travel burden has been found to influence the choice of treatment and treatment site, to lower treatment adherence and to worsen prognosis 49, 50. There is also convincing evidence that quality of CRC surgery depends on hospital volume and surgeon's clinical experience 51, 52. Therefore, observed survival disparities for CRC might be at least partly explained by differences in quality of care, assuming that patients with cancer living in rural areas are treated more frequently at low‐volume hospitals located closest to their home 50.

Compared to Swiss nationals, survival of non‐Swiss patients with CRC was poorer, even after adjustment for stage at diagnosis, SEP, and further sociodemographic characteristics. Interestingly, patients' nationality had no impact on CRC stage at diagnosis, suggesting equal access to screening and diagnostic procedures. The difference in CRC survival between Swiss and foreigners might be due to adherence to treatment, delay in treatment, or other unmeasured factors and warrants further studies. The non‐Swiss population is composed of highly heterogeneous groups according to the country of origin, migration status (first‐, second‐, or third‐generation immigrants), type of residence permit, income, employment, and knowledge of one of the Swiss national languages**,** to name a few. Further investigations of this issue should pay particular attention to the broad diversity of immigrants and foreigners living in Switzerland. This is especially important as there is a lack of information about migrants' health and health needs in Switzerland.

Finally, as observed in other studies, patients with rectal cancer compared with patients with colon cancer showed a reduced risk of being diagnosed at later stages 19, 20. This pattern by anatomic site might reflect the effect of screening which is more effective for distal than proximal subsites 9. Stage‐adjusted survival was lower in patients with rectal cancer than colon cancer, which might be related to etiological differences and differences in treatment response 53.

### Strengths and limitations

This study is the first population‐based Swiss study investigating socioeconomic inequalities in CRC stage at diagnosis and subsequent survival. With the exception of the Geneva CR, information on education and other SEP indicators is not systematically collected by other Swiss CRs. The dataset of the SNC‐NICER Cancer Epidemiology Study opened the opportunity, for the first time, to investigate this topic using data from multiple Swiss cantons.

The seven participating CRs covered approximately 50% of the Swiss population. The covered population was not representative of the Swiss population with regard to SEP, nationality, urbanity, and language region of residence. Therefore, generalizability of these findings is limited.

Our study has some limitations. First, SEP is a multidimensional construct and highest completed education level, which has been used as proxy for SEP in our study, does not fully capture it. However, education forms a unique dimension of SEP, with qualities that make it especially important to health 54. Further, education precedes and influences other dimensions of SEP, such as occupational status and personal income 54. In addition, there is considerable international evidence that education is strongly associated with health, health behavior, and preventive service use and that a substantial share of these effects is of causal origin 55. In addition, education is fairly stable after early adulthood and virtually complete in the study population (>99%).

Another drawback of the study is the lack of more detailed tumor characteristics (besides TNM stage) and other prognostic factors, such as comorbidities and CRC treatment. TNM stage is the most important single predictor for CRC survival. However, TNM stage information of CRC provides the strongest prognostic information for patients with early or advanced disease, but is of limited value for intermediate stages 2. For example, survival of stage IIB tumors is lower than that of some stage III CRCs. Therefore, Doubeni et al. 56, for example, defined late stage as stage IIB‐IV CRCs. Due to the lack of histopathologic subtype information, this was not feasible in our study, but calculations have been redone using alternative cutoffs (I‐II vs. III‐IV and I‐III vs. IV). Overall, the knowledge of additional prognostic tumor characteristics such as histopathologic subtype, grade, lymphatic invasion, venous invasion, or perineural invasion 2 would have particularly improved our risk adjustment for patients diagnosed with intermediate stages.

In patients with CRC, comorbidities are common, particularly in older males 57, 58 with low SEP 57. Comorbidities have an adverse effect on overall and stage‐specific CRC survival 59, 60. Furthermore, patients with comorbidities are less likely to receive standard therapy and to complete treatment courses as intended 59. However, whether these treatment disparities reflect appropriate treatment decisions of the treating physicians, patient preferences, poorer treatment adherence of the patients, or inequalities in cancer care remains unclear 59 and needs individual investigations by country/region and healthcare system. Unfortunately, comorbidity status of the patients was not available for this study. In addition, no individual data concerning CRC screening history was available for this study.

In addition, misclassification of cause of death might have biased survival estimates 61. Therefore, performing relative survival would have been beneficial, also in respect of potential counfounding by comorbidity status (see above). However, due to the lack of adequate life tables (e.g., life stables stratified by socioeconomic position.), this was not feasible within this study.

Finally, sociodemographic characteristics (except age at diagnosis) were obtained from the census. With increasing time between date of census and end of follow‐up, characteristics such as marital status or place of residence might have changed resulting in misclassification when referring to the time of or after CRC diagnosis.

## Conclusions

In Switzerland, the majority of patients with CRC are still diagnosed at a late stage, and the likelihood to have a late‐stage CRC is highest in people of low SEP. These findings highlight the need to increase screening prevalence and awareness of the benefits of CRC screening in all social groups. As the net benefit of CRC screening is well documented, the implementation of organized population‐based CRC screening programs in all cantons of Switzerland should be a priority of Swiss healthcare policies. Further, exempting CRC screening from the franchise and the deductible of the basic health insurance might help to especially increase the screening prevalence in the population with lower SEP.

Survival inequalities by SEP could be sufficiently explained by differences in stage at diagnosis, arguing against substantial socioeconomic inequalities in CRC treatment. But importantly, non‐Swiss compared to Swiss citizens and patients living in nonurban areas compared to their urban counterparts showed poorer survival even in the fully adjusted model, suggesting that differences in treatment adherence or quality of care might play a role. Reasons underpinning these inequalities, however, warrant further investigations in order to identify factors that can be addressed to improve outcomes equally in all social groups.

Overall, it is alarming that these social inequalities have been observed in Switzerland, a wealthy country with universal health insurance coverage and one of the highest life expectancies in the world. Swiss public health strategies should facilitate equal access to CRC screening and optimal CRC care for all social groups and in all regions of Switzerland.

## Conflict of Interest

None declared.

## Supporting information


**Table S1.** Distribution of socioeconomic and demographic characteristics of the cantons participating in the study and all Switzerland (population aged 30–84 years), Census 2000.
**Table S2.** Contribution of colorectal cancer cases and person‐years by cancer registry: incidence period 05/12/2000 ‐ 31/12/2008, patients aged 30–84 years at diagnosis.
**Table S3.** Odds ratios (OR) of later colorectal cancer stage at diagnosis (Stage I‐II vs. Stage III‐IV).
**Table S4.** Odds ratios (OR) of later colorectal cancer stage at diagnosis (Stage I‐III vs. Stage IV).
**Table S5.** Cause‐specific hazard ratios (CHR) in patients with colorectal cancer.
**Table S6.** Odds ratios (OR) of later colorectal cancer stage at diagnosis after multiple imputation of stage.
**Table S7.** Sub‐hazard ratios and 95% confidence intervals (95% CI), risk of colorectal cancer death in colorectal cancer patients after multiple imputation of stage.
