# Supplementary material for: Socioeconomic and demographic inequalities in stage at diagnosis and survival among colorectal cancer patients: evidence from a Swiss population‐based study
Source: Cancer Med. 2018 Feb 26;7(4):1498–510. doi: 10.1002/cam4.1385 (PMC5911574; doi:10.1002/cam4.1385)
Supplement: Supplementary file 1 — Table S1. Distribution of socioeconomic and demographic characteristics of the cantons participating in the study and all Switzerland (population aged 30–84 years), Census 2000. Table S2. Contribution of colorectal cancer cases and person‐years by cancer registry: incidence period 05/12/2000 ‐ 31/12/2008, patients aged 30–84 years at diagnosis. Table S3. Odds ratios (OR) of later colorectal cancer stage at diagnosis (Stage I‐II vs. Stage III‐IV). Table S4. Odds ratios (OR) of later colorectal cancer stage at diagnosis (Stage I‐III vs. Stage IV). Table S5. Cause‐specific hazard ratios (CHR) in patients with colorectal cancer. Table S6. Odds ratios (OR) of later colorectal cancer stage at diagnosis after multiple imputation of stage. Table S7. Sub‐hazard ratios and 95% confidence intervals (95% CI), risk of colorectal cancer death in colorectal cancer patients after multiple imputation of stage. [file CAM4-7-1498-s001.docx]

**APPENDIX**

**Suppl. Table 1:** Distribution of socioeconomic and demographic characteristics of the cantons participating in the study and all Switzerland (population aged 30-84 years), Census 2000

| **Socioeconomic and demographic  characteristics** | **Cantons participating in the study** | | **Whole of   Switzerland** | |
| --- | --- | --- | --- | --- |
|  | N | column % | N | column % |
|  |  |  |  |  |
| **Socioeconomic position (SEP)** |  |  |  |  |
| low SEP | 489,870 | 23.8 | 1,078,424 | 23.7 |
| middle SEP | 999,265 | 48.5 | 2,331,895 | 51.3 |
| high SEP | 507,472 | 24.6 | 1,010,800 | 22.2 |
| unknown | 64,338 | 3.1 | 124,213 | 2.7 |
|  |  |  |  |  |
| **Sex** |  |  |  |  |
| male | 991,467 | 48.1 | 2,202,279 | 48.5 |
| female | 1,069,478 | 51.9 | 2,343,053 | 51.6 |
|  |  |  |  |  |
| **Age at diagnosis** |  |  |  |  |
| 30-49 years | 1,039,029 | 50.4 | 2,287,496 | 50.3 |
| 50-64 years | 590,850 | 28.7 | 1,288,317 | 28.3 |
| 65-74 years | 284,981 | 13.8 | 637,993 | 14.0 |
| 75-84 years | 146,085 | 7.1 | 331,526 | 7.3 |
|  |  |  |  |  |
| **Civil status** |  |  |  |  |
| single | 321,193 | 15.6 | 683,058 | 15.0 |
| married | 1,393,206 | 67.6 | 3,134,345 | 69.0 |
| widowed | 143,630 | 7.0 | 323,982 | 7.1 |
| divorced | 202,916 | 9.9 | 403,947 | 8.9 |
|  |  |  |  |  |
| **Urbanity of residence** |  |  |  |  |
| urban | 712,110 | 34.6 | 1,309,476 | 28.8 |
| peri-urban | 1,021,000 | 49.5 | 2,059,108 | 45.3 |
| rural | 327,835 | 15.9 | 1,176,748 | 25.9 |
|  |  |  |  |  |
| **Language-region** |  |  |  |  |
| German-speaking region | 877,042 | 42.6 | 3,278,361 | 72.1 |
| French-speaking region | 980,809 | 47.6 | 1,054,883 | 23.2 |
| Italian-speaking region | 203,094 | 9.9 | 212,088 | 4.7 |
|  |  |  |  |  |
| **Nationality** |  |  |  |  |
| Swiss | 1,585,929 | 77.0 | 3,700,941 | 81.4 |
| non-Swiss | 475,016 | 23.1 | 844,391 | 18.6 |
|  |  |  |  |  |
| **Total N row %** | **2,060,945** | **45.3** | **4,545,332** | **100.0** |

**Suppl. Table 2:** Contribution of colorectal cancer cases and person-years by cancer registry: incidence period 05/12/2000 - 31/12/2008, patients aged 30-84 years at diagnosis

| CR | all stages | | |  | with stage information | | |
| --- | --- | --- | --- | --- | --- | --- | --- |
|  | Cases  (N) | Person-years (PY) of follow-up | % of pooled PY |  | Cases  (N) | Person-years (PY) of follow-up | % of pooled PY |
| Fribourg | 294 | 1,344 | 2.4 |  | 277 | 1,273 | 2.4 |
| Geneva | 1,214 | 7,250 | 12.8 |  | 1,147 | 7,016 | 13.4 |
| Neuchâtel | 601 | 3,284 | 5.8 |  | 540 | 3,125 | 6.0 |
| Ticino | 1,276 | 7,216 | 12.7 |  | 1,224 | 6,967 | 13.3 |
| Valais | 910 | 5,043 | 8.9 |  | 855 | 4,771 | 9.1 |
| Vaud | 2,021 | 11,754 | 20.7 |  | 1,704 | 10,064 | 19.2 |
| Zurich | 3,772 | 20,766 | 36.7 |  | 3,400 | 19.298 | 36.7 |

Note: 82 cases (0.8%) out of originally 10.170 cases aged 30-84 years have been excluded due to missing SEP information. From the remaining dataset (N=10,088), 140 (1.4%) additional cases were excluded for survival analyses due to zero survival time (death certificate only cases or cases first diagnosed at autopsy).

**Suppl. Table 3**: Odds ratios (OR) of later colorectal cancer stage at diagnosis (Stage I-II versus Stage III-IV)

|  | **Model 1** | | **Model 2** | | **Model 3** | | **Model 4** | | **Model 5*** | |
| --- | --- | --- | --- | --- | --- | --- | --- | --- | --- | --- |
|  | OR | [95%CI] | OR | [95%CI] | OR | [95%CI] | OR | [95%CI] | OR | [95%CI] |
|  |  |  |  |  |  |  |  |  |  |  |
| **SEP** |  |  |  |  |  |  |  |  |  |  |
| High SEP (ref.) |  |  |  |  |  |  |  |  |  |  |
| Middle SEP | 1.00 | [0.90-1.12] | 1.00 | [0.90-1.11] | 1.00 | [0.90-1.12] | 1.00 | [0.90-1.12] | 0.99 | [0.90-1.11] |
| Low SEP | 1.05 | [0.94-1.19] | 1.05 | [0.93-1.19] | 1.05 | [0.92-1.19] | 1.05 | [0.92-1.19] | 1.04 | [0.92-1.19] |
|  |  |  |  |  |  |  |  |  |  |  |
| **Sex** |  |  |  |  |  |  |  |  |  |  |
| male (ref.) |  |  |  |  |  |  |  |  |  |  |
| female |  |  | 1.12 | [1.03-1.23] | 1.12 | [1.03-1.23] | 1.12 | [1.03-1.23] | 1.12 | [1.03-1.23] |
|  |  |  |  |  |  |  |  |  |  |  |
| **Age at diagnosis** | |  |  |  |  |  |  |  |  |  |
| 50-64 years (ref.) | | |  |  |  |  |  |  |  |  |
| < 50 years |  |  | 1.36 | [1.14-1.63] | 1.37 | [1.14-1.64] | 1.36 | [1.14-1.64] | 1.37 | [1.14-1.64] |
| 65-74 years |  |  | 0.95 | [0.85-1.06] | 0.95 | [0.86-1.06] | 0.95 | [0.86-1.06] | 0.95 | [0.86-1.06] |
| 75-84 years |  |  | 0.89 | [0.80-1.00] | 0.89 | [0.80-1.00] | 0.89 | [0.80-1.00] | 0.89 | [0.80-1.00] |
|  |  |  |  |  |  |  |  |  |  |  |
| **Civil status** |  |  |  |  |  |  |  |  |  |  |
| married (ref.) |  |  |  |  |  |  |  |  |  |  |
| single |  |  | 1.32 | [1.13-1.53] | 1.31 | [1.13-1.53] | 1.31 | [1.13-1.53] | 1.31 | [1.13-1.53] |
| widowed |  |  | 0.94 | [0.82-1.07] | 0.94 | [0.82-1.06] | 0.94 | [0.82-1.07] | 0.94 | [0.82-1.07] |
| divorced |  |  | 1.09 | [0.95-1.25] | 1.08 | [0.94-1.25] | 1.08 | [0.94-1.25] | 1.09 | [0.95-1.25] |
|  |  |  |  |  |  |  |  |  |  |  |
| **Nationality** |  |  |  |  |  |  |  |  |  |  |
| Swiss (ref.) |  |  |  |  |  |  |  |  |  |  |
| Non-Swiss |  |  | 1.07 | [0.96-1.20] | 1.07 | [0.96-1.20] | 1.07 | [0.96-1.20] | 1.07 | [0.96-1.21] |
|  |  |  |  |  |  |  |  |  |  |  |
| **Urbanity** |  |  |  |  |  |  |  |  |  |  |
| urban (ref.) |  |  |  |  |  |  |  |  |  |  |
| peri-urban |  |  |  |  | 0.98 | [0.89-1.07] | 0.98 | [0.89-1.07] | 0.98 | [0.89-1.07] |
| rural |  |  |  |  | 1.01 | [0.88-1.15] | 1.00 | [0.88-1.15] | 1.00 | [0.87-1.15] |
|  |  |  |  |  |  |  |  |  |  |  |
| **Language region**** | |  |  |  |  |  |  |  |  |  |
| German (ref.) |  |  |  |  |  |  |  |  |  |  |
| French |  |  |  |  | 1.03 | [0.94-1.12] | 1.03 | [0.94-1.12] | 0.99 | [0.76-1.31] |
| Italian |  |  |  |  | 0.97 | [0.85-1.10] | 0.97 | [0.85-1.10] | 0.98 | [0.86-1.11] |
|  |  |  |  |  |  |  |  |  |  |  |
| **Localisation** |  |  |  |  |  |  |  |  |  |  |
| Colon (ref.) |  |  |  |  |  |  |  |  |  |  |
| rectum |  |  |  |  |  |  | 0.99 | [0.90.1.08] | 0.99 | [0.90-1.08] |

* Additionately adjusted for canton of residence to account for unmeasured canton characteristics associated with SEP and stage.

**German-speaking region: eastern parts of the canton of Fribourg, eastern parts of the canton of Valais (upper Valais), canton of Zurich; French-speaking region: western parts of the canton of Fribourg, canton of Geneva, canton of Neuchâtel, western parts of the canton of Valais (Central and Lower Valais), canton of Vaud; Italian-speaking region: canton of Ticino.

**Suppl. Table 4**: Odds ratios (OR) of later colorectal cancer stage at diagnosis (Stage I-III versus Stage IV)

|  | **Model 1** | | **Model 2** | | **Model 3** | | **Model 4** | | **Model 5*** | |
| --- | --- | --- | --- | --- | --- | --- | --- | --- | --- | --- |
|  | OR | [95%CI] | OR | [95%CI] | OR | [95%CI] | OR | [95%CI] | OR | [95%CI] |
|  |  |  |  |  |  |  |  |  |  |  |
| **SEP** |  |  |  |  |  |  |  |  |  |  |
| High SEP (ref.) |  |  |  |  |  |  |  |  |  |  |
| Middle SEP | 1.03 | [0.91-1.18] | 1.05 | [0.92-1.20] | 1.05 | [0.92-1.20] | 1.06 | [0.92-1.21] | 1.05 | [0.92-1.20] |
| Low SEP | 1.00 | [0.86-1.15] | 1.02 | [0.88-1.19] | 1.00 | [0.86-1.17] | 1.01 | [0.87-1.18] | 0.98 | [0.84-1.15] |
|  |  |  |  |  |  |  |  |  |  |  |
| **Sex** |  |  |  |  |  |  |  |  |  |  |
| male (ref.) |  |  |  |  |  |  |  |  |  |  |
| female |  |  | 1.02 | [0.92-1.14] | 1.03 | [0.92-1.15] | 1.01 | [0.90-1.12] | 1.02 | [0.91-1.14] |
|  |  |  |  |  |  |  |  |  |  |  |
| **Age at diagnosis** | |  |  |  |  |  |  |  |  |  |
| 50-64 years (ref.) | | |  |  |  |  |  |  |  |  |
| < 50 years |  |  | 1.20 | [0.98-1.48] | 1.21 | [0.99-1.49] | 1.21 | [0.98-1.48] | 1.21 | [0.99-1.49] |
| 65-74 years |  |  | 0.93 | [0.82-1.06] | 0.94 | [0.82-1.06] | 0.92 | [0.81-1.05] | 0.93 | [0.81-1.05] |
| 75-84 years |  |  | 0.92 | [0.80-1.05] | 0.92 | [0.80-1.05] | 0.89 | [0.77-1.02] | 0.90 | [0.78-1.03] |
|  |  |  |  |  |  |  |  |  |  |  |
| **Civil status** |  |  |  |  |  |  |  |  |  |  |
| married (ref.) |  |  |  |  |  |  |  |  |  |  |
| single |  |  | 1.25 | [1.05-1.48] | 1.24 | [1.04-1.47] | 1.24 | [1.04-1.48] | 1.25 | [1.05-1.49] |
| widowed |  |  | 0.90 | [0.76-1.07] | 0.89 | [0.76-1.06] | 0.90 | [0.76-1.06] | 0.90 | [0.76-1.07] |
| divorced |  |  | 1.21 | [1.03-1.42] | 1.20 | [1.03-1.42] | 1.21 | [1.02-1.42] | 1.23 | [1.04-1.45] |
|  |  |  |  |  |  |  |  |  |  |  |
| **Nationality** |  |  |  |  |  |  |  |  |  |  |
| Swiss (ref.) |  |  |  |  |  |  |  |  |  |  |
| Non-Swiss |  |  | 1.09 | [0.95-1.24] | 1.07 | [0.94-1.23] | 1.07 | [0.93-1.23] | 1.09 | [0.95-1.25] |
|  |  |  |  |  |  |  |  |  |  |  |
| **Urbanity** |  |  |  |  |  |  |  |  |  |  |
| urban (ref.) |  |  |  |  |  |  |  |  |  |  |
| peri-urban |  |  |  |  | 0.95 | [0.85-1.07] | 0.95 | [0.85-1.07] | 0.96 | [0.86-1.08] |
| rural |  |  |  |  | 0.99 | [0.85-1.16] | 0.98 | [0.83-1.15] | 0.92 | [0.78-1.09] |
|  |  |  |  |  |  |  |  |  |  |  |
| **Language region**** | |  |  |  |  |  |  |  |  |  |
| German (ref.) |  |  |  |  |  |  |  |  |  |  |
| French |  |  |  |  | 1.15 | [1.03-1.28] | 1.14 | [1.02-1.28] | 1.03 | [0.76-1.41] |
| Italian |  |  |  |  | 1.18 | [1.01-1.38] | 1.16 | [0.99-1.36] | 1.21 | [1.03-1.42] |
|  |  |  |  |  |  |  |  |  |  |  |
| **Localisation** |  |  |  |  |  |  |  |  |  |  |
| Colon (ref.) |  |  |  |  |  |  |  |  |  |  |
| rectum |  |  |  |  |  |  | 0.74 | [0.66-0.82] | 0.74 | [0.66-0.83] |

* Additionately adjusted for canton of residence to account for unmeasured canton characteristics associated with SEP and stage.

**German-speaking region: eastern parts of the canton of Fribourg, eastern parts of the canton of Valais (upper Valais), canton of Zurich; French-speaking region: western parts of the canton of Fribourg, canton of Geneva, canton of Neuchâtel, western parts of the canton of Valais (Central and Lower Valais), canton of Vaud; Italian-speaking region: canton of Ticino.

**Suppl. Table 5.** Cause-specific hazard ratios (CHR) in patients with colorectal cancer

|  | **colorectal cancer  death** | | **death due to other causes** | |
| --- | --- | --- | --- | --- |
|  | CHR | [95%CI] | CHR | [95%CI] |
|  |  |  |  |  |
| **SEP** |  |  |  |  |
| High SEP (ref.) |  |  |  |  |
| Middle SEP | 1.01 | [0.92-1.12] | 1.16 | [1.00-1.34] |
| Low SEP | 1.12 | [1.00-1.25] | 1.24 | [1.02-1.71] |
|  |  |  |  |  |
| **Sex** |  |  |  |  |
| male (ref.) |  |  |  |  |
| female | 0.93 | [.0.86-1.01] | 0.65 | [0.58-0.73] |
|  |  |  |  |  |
| **Age at diagnosis** | |  |  |  |
| 50-64 years (ref.) | | |  |  |
| < 50 years | 0.68 | [0.57-0.81] | 0.47 | [30.38-72.03] |
| 65-74 years | 1.25 | [1.13-1.37] | 2.29 | [1.94-1.21] |
| 75-84 years | 2.00 | [1.81-2.20] | 15.48 | [1.35-1.62] |
|  |  |  |  |  |
| **Civil status** |  |  |  |  |
| married (ref.) |  |  |  |  |
| single | 1.04 | [0.92-1.19] | 1.14 | [1.00-1.29] |
| widowed | 0.99 | [0.88-1.11] | 0.89 | [0.79-1.00] |
| divorced | 1.00 | [0.88-1.13] | 1.10 | [0.98-1.24] |
|  |  |  |  |  |
| **Nationality** |  |  |  |  |
| Swiss (ref.) |  |  |  |  |
| Non-Swiss | 1.23 | [1.10-1.36] | 1.15 | [1.04-1.27] |
|  |  |  |  |  |
| **Urbanity** |  |  |  |  |
| urban (ref.) |  |  |  |  |
| peri-urban | 1.16 | [1.07-1.26] | 1.04 | [0.93-1.16] |
| rural | 1.15 | [1.02-1.30] | 1.01 | [0.85-1.19] |
|  |  |  |  |  |
| **Language region*** | |  |  |  |
| German (ref.) |  |  |  |  |
| French | 0.96 | [0.77-1.20] | 1.00 | [0.70-1.43] |
| Italian | 0.93 | [0.82-1.04] | 1.02 | [0.88-1.19] |
|  |  |  |  |  |
| **Localisation** |  |  |  |  |
| Colon (ref.) |  |  |  |  |
| rectum | 1.11 | [1.02-1.19] | 1.01 | [0.91-1.13] |
|  |  |  |  |  |
| Stage at diagnosis |  |  |  |  |
| Stage I (ref.) |  |  |  |  |
| Stage II | 3.24 | [2.57-4.08] | 1.08 | [0.95-1.24] |
| Stage III | 8.34 | [6.70-10.39] | 1.03 | [0.89-1.19] |
| Stage IV | 35.50 | [28.54-44.16] | 2.12 | [1.77-2.53] |

^1^*German-speaking region: eastern parts of the canton of Fribourg, eastern parts of the canton of Valais (upper Valais), canton of Zurich; French-speaking region: western parts of the canton of Fribourg, canton of Geneva, canton of Neuchâtel, western parts of the canton of Valais (Central and Lower Valais), canton of Vaud; Italian-speaking region: canton of Ticino.

**Suppl. Table 6**: Odds ratios (OR) of later colorectal cancer stage at diagnosis after multiple imputation of stage

|  | **Stage I versus  Stage II-IV** | | **Stage I-II versus Stage II-IV** | | **Stage I-III versus Stage IV** | |
| --- | --- | --- | --- | --- | --- | --- |
|  | OR | [95%CI] | OR | [95%CI] | OR | [95%CI] |
|  |  |  |  |  |  |  |
| **SEP** |  |  |  |  |  |  |
| High SEP (ref.) |  |  |  |  |  |  |
| Middle SEP | 1.11 | [0.97-1.28] | 1.01 | [0.90-1.12] | 1.06 | [0.92-1.21] |
| Low SEP | 1.29 | [1.10-1.52] | 1.06 | [0.93-1.20] | 1.00 | [0.85-1.16] |
|  |  |  |  |  |  |  |
| **Sex** |  |  |  |  |  |  |
| male (ref.) |  |  |  |  |  |  |
| Female | 1.05 | [0.93-1.18] | 1.12 | [1.02-1.22] | 1.01 | [0.91-1.13] |
|  |  |  |  |  |  |  |
| **Age at diagnosis** | |  |  |  |  |  |
| 50-64 years (ref.) | | |  |  |  |  |
| < 50 years | 1.16 | [0.92-1.48] | 1.32 | [1.11-1.58] | 1.18 | [0.97-1.44] |
| 65-74 years | 1.02 | [0.89-1.17] | 0.95 | [0.86-1.06] | 0.94 | [0.83-1.07] |
| 75-84 years | 1.18 | [1.02-1.37] | 0.93 | [0.83-1.04] | 0.97 | [0.84-1.11] |
|  |  |  |  |  |  |  |
| **Civil status** |  |  |  |  |  |  |
| married (ref.) |  |  |  |  |  |  |
| Single | 1.37 | [1.11-1.68] | 1.34 | [1.15-1.55] | 1.31 | [1.10-1.55] |
| Widowed | 1.05 | [0.87-1.25] | 0.94 | [0.82-1.07] | 0.89 | [0.75-1.06] |
| Divorced | 1.17 | [0.97-1.41] | 1.11 | [0.97-1.27] | 1.26 | [1.07-1.48] |
|  |  |  |  |  |  |  |
| **Nationality** |  |  |  |  |  |  |
| Swiss (ref.) |  |  |  |  |  |  |
| Non-Swiss | 1.02 | [0.88-1.19] | 1.07 | [0.95-1.20] | 1.08 | [0.94-1.23] |
|  |  |  |  |  |  |  |
| **Urbanity** |  |  |  |  |  |  |
| urban (ref.) |  |  |  |  |  |  |
| peri-urban | 0.93 | [0.82-1.04] | 0.97 | [0.89-1.07] | 0.96 | [0.86-1.07] |
| Rural | 0.96 | [0.80-1.15] | 1.00 | [0.87-1.14] | 0.93 | [0.79-1.09] |
|  |  |  |  |  |  |  |
| **Language region** | |  |  |  |  |  |
| German (ref.) |  |  |  |  |  |  |
| French | 1.16 | [0.82-1.66] | 0.99 | [0.75-1.30] | 1.03 | [0.76-1.40] |
| Italian | 0.92 | [0.78-1.09] | 0.96 | [0.84-1.10] | 1.16 | [0.99-1.36] |
|  |  |  |  |  |  |  |
| **Localisation** |  |  |  |  |  |  |
| Colon (ref.) |  |  |  |  |  |  |
| Rectum | 0.68 | [0.61-0.76] | 0.99 | [0.91-1.08] | 0.74 | [0.67-0.83] |

UICC stage was imputed with 25 imputations using the following variables as predictors: age at diagnosis and follow-up time as continuous predictors, SEP, sex, civil status, nationality, urbanity, language region, tumour localisation and follow-up status (alive, death due to CRC, other cause of death) as categorical predictors[^32^](#_ENREF_32), and an interaction term between follow-up time and follow-up status[^33^](#_ENREF_33).

All models have been additionally adjusted for canton of residence.

^1^ German-speaking region: eastern parts of the canton of Fribourg, eastern parts of the canton of Valais (upper Valais), canton of Zurich; French-speaking region: western parts of the canton of Fribourg, canton of Geneva, canton of Neuchâtel, western parts of the canton of Valais (Central and Lower Valais), canton of Vaud; Italian-speaking region: canton of Ticino.

**Suppl. Table 7:** Sub-hazard ratios and 95% confidence intervals (95%CI), risk of colorectal cancer death in colorectal cancer patients after multiple imputation of stage

|  | **Final Model** | |
| --- | --- | --- |
|  | SHR | [95%CI] |
|  |  |  |
| **SEP** |  |  |
| High SEP (ref.) |  |  |
| Middle SEP | 1.00 | [0.91-1.11] |
| Low SEP | 1.10 | [0.99-1.24] |
|  |  |  |
| **Sex** |  |  |
| male (ref.) |  |  |
| female | 0.96 | [0.88-1.04] |
|  |  |  |
| **Age at diagnosis** | |  |
| 50-64 years (ref.) | | |
| < 50 years | 0.69 | [0.58-0.81] |
| 65-74 years | 1.19 | [1.08-1.30] |
| 75-84 years | 1.70 | [1.54-1.87] |
|  |  |  |
| **Civil status** |  |  |
| married (ref.) |  |  |
| single | 1.01 | [0.88-1.15] |
| widowed | 0.96 | [0.85-1.08] |
| divorced | 1.03 | [0.91-1.16] |
|  |  |  |
| **Nationality** |  |  |
| Swiss (ref.) | 1.20 | [1.09-1.34] |
| Non-Swiss |  |  |
|  |  |  |
| **Urbanity** |  |  |
| urban (ref.) |  |  |
| peri-urban | 1.13 | [1.04-1.23] |
| rural | 1.18 | [1.05-1.32] |
|  |  |  |
| **Language region*** | |  |
| German (ref.) |  |  |
| French | 0.96 | [0.77-1.20] |
| Italian | 0.90 | [0.80-1.02] |
|  |  |  |
| **Localisation** |  |  |
| Colon (ref.) |  |  |
| rectum | 1.11 | [1.03-1.20] |
|  |  |  |
| **Stage at diagnosis** |  |  |
| Stage I (ref.) |  |  |
| Stage II | 2.88 | [2.31-3.60] |
| Stage III | 7.05 | [5.69-8.73] |
| Stage IV | 24.93 | [20.17-30.80] |

UICC stage was imputed with 25 imputations using the following variables as predictors: age at diagnosis and follow-up time as continuous predictors, SEP, sex, civil status, nationality, urbanity, language region, tumour localisation and d follow-up status (alive, death due to CRC, other cause of death) as categorical predictors[^32^](#_ENREF_32), and an interaction term between follow-up time and follow-up status[^33^](#_ENREF_33).

Survival was analysed using competing risk regressions based on Fine and Gray's proportional hazard model [^30^](#_ENREF_30). All underlying causes of death other than colorectal cancer (CRC) were classified as competing risks. The model has been adjusted for all variables listed plus canton of residence. .Results are reported as sub-hazard ratios for risk of dying due to CRC (SHRs) with 95% confidence intervals (95%CI).

^*^German-speaking region: eastern parts of the canton of Fribourg, eastern parts of the canton of Valais (upper Valais), canton of Zurich; French-speaking region: western parts of the canton of Fribourg, canton of Geneva, canton of Neuchâtel, western parts of the canton of Valais (Central and Lower Valais), canton of Vaud; Italian-speaking region: canton of Ticino.
